# A role for pancreatic beta-cell secretory hyperresponsiveness in catch-up growth hyperinsulinemia: *Relevance to thrifty catch-up fat phenotype and risks for type 2 diabetes*

**DOI:** 10.1186/1743-7075-8-2

**Published:** 2011-01-18

**Authors:** Marina Casimir, Paula B de Andrade, Asllan Gjinovci, Jean-Pierre Montani, Pierre Maechler, Abdul G Dulloo

**Affiliations:** 1 Department of Medicine / Physiology, University of Fribourg, Switzerland; 2 Department of Cell Physiology and Metabolism, University of Geneva, Switzerland; 3 Department of Medicine / Physiology, University of Fribourg, Rue du Musée 5, CH-1700 Fribourg, Switzerland

## Abstract

Current notions about mechanisms by which catch-up growth predisposes to later type 2 diabetes center upon those that link hyperinsulinemia with an accelerated rate of fat deposition (catch-up fat). Using a rat model of semistarvation-refeeding in which catch-up fat is driven solely by elevated metabolic efficiency associated with hyperinsulinemia, we previously reported that insulin-stimulated glucose utilization is diminished in skeletal muscle but increased in white adipose tissue. Here, we investigated the possibility that hyperinsulinemia during catch-up fat can be contributed by changes in the secretory response of pancreatic beta-cells to glucose. Using the rat model of semistarvation-refeeding showing catch-up fat and hyperinsulinemia, we compared isocalorically refed and control groups for potential differences in pancreatic morphology and in glucose-stimulated insulin secretion during *in situ *pancreas perfusions as well as *ex vivo *isolated islet perifusions. Between refed and control animals, no differences were found in islet morphology, insulin content, and the secretory responses of perifused isolated islets upon glucose stimulation. By contrast, the rates of insulin secretion from *in situ *perfused pancreas showed that raising glucose from 2.8 to 16.7 mmol/l produced a much more pronounced increase in insulin release in refed than in control groups (p < 0.01). These results indicate a role for islet secretory hyperresponsiveness to glucose in the thrifty mechanisms that drive catch-up fat through glucose redistribution between skeletal muscle and adipose tissue. Such beta-cell hyperresponsiveness to glucose may be a key event in the link between catch-up growth, hyperinsulinemia and risks for later type 2 diabetes.

## Introduction

A large body of evidence indicate that subjects who had low birth weight or who showed reduced growth rate during childhood, but who subsequently showed catch-up growth, have higher susceptibility for type 2 diabetes or cardiovascular diseases later in life [1-5]. While the nature of this association between catch-up growth and later disease risks remains obscure [6], it is intricately linked to the state of hyperinsulinemia and accelerated recovery of body fat (catch-up fat) that characterizes catch-up growth [5-7]. There is a well-described rat model of semistarvation-refeeding in which catch-up fat and hyperinsulinemia occur in absence of hyperphagia and could be linked to an elevated metabolic efficiency due to suppressed thermogenesis [8]. Using this model, we previously showed that insulin-mediated glucose utilization is diminished in skeletal muscle but enhanced in white adipose tissue [9], thereby suggesting that catch-up fat is characterized by glucose redistribution from skeletal muscle to adipose tissue. The suppressed thermogenesis is thus associated with establishment of a thrifty metabolism which spares glucose for catch-up fat *via *coordinated induction of insulin resistance in skeletal muscle, insulin hyperresponsiveness in adipose tissue and a state of hyperinsulinemia. In this context, putative implication of insulin-secreting cells remains unknown. Here, we tested the hypothesis that the hyperinsulinemic state of catch-up fat might also be contributed by pancreatic beta-cell hyperresponsiveness to glucose. To this end, we investigated the semistarvation-refeeding rat model for pancreatic endocrine function and morphology. In particular, the secretory responses of perfused pancreases and isolated islets were analyzed.

## Methods

### Animals and Diet

Male Sprague Dawley rats (Elevage Janvier, France), caged singly in a temperature-controlled room (22 ± 1°C) with 12-h light/dark cycle, were maintained on chow diet (Kliba, Cossonay, Switzerland) consisting, by energy, of 24% protein, 66% carbohydrates, and 10% fat, and had free access to tap water. Animals were maintained in accordance with our institute's regulations and guide for the care and use of laboratory animals.

### Design of study

**Figure 1:**
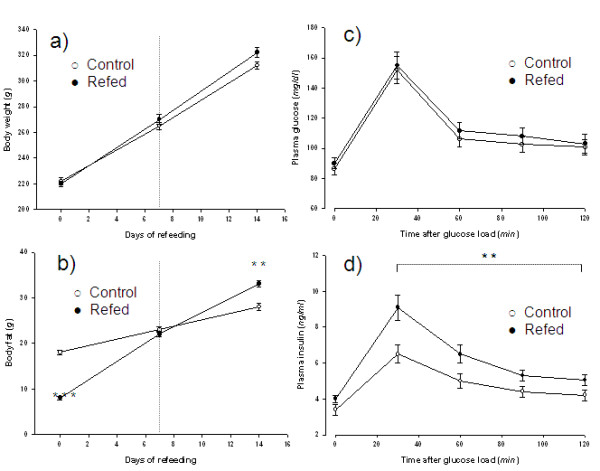
**Rat model of catch-up fat and hyperinsulinemia**. Panels a and b show data (mean ± SE, n = 6) for body weight and body fat, respectively, at the end of semistarvation (corresponding to day 0 of refeeding), and at day 7 and 14 of refeeding in refed and control groups consuming isocaloric amounts of food. After sacrifice, the whole carcasses were dried to a constant weight in an oven maintained at 70°C and subsequently homogenized for analysis of fat content by the Soxhlet extraction method as previously described (8). No between-group differences are found in dry lean tissue mass at all time-points; ** p < 0.01; *** p < 0.001. Panels c and d show data (mean ± SE, n = 6) for plasma glucose and insulin before and after an intraperitoneal glucose tolerance test (GTT), which was performed as previously described (8) on day 7 of refeeding, by ip administration of 2 g glucose /kg body weight. Plasma glucose was determined using a Beckman glucose analyzer, while plasma insulin was assessed using rat insulin ELISA kit (Crystal Chem Inc, IL, USA); **: p < 0.01

The experimental design is similar to that previously described [8]. Seven wk old rats were food-restricted at 50% of their spontaneous food intake for 2 wks, after which they were refed the same amount of chow corresponding to spontaneous chow intake of control rats matched for weight at the onset of refeeding. Under these conditions, the refed animals show similar gain in lean mass, but 2-fold greater fat gain than controls, due to 10-13% lower energy expenditure resulting from suppressed thermogenesis [8]. Pancreatic function was assessed on day 7 of refeeding, i.e. at a time-point when, as shown in Figure 1, body fat in refed animals has not yet exceeded that of controls, and when refed animals showing catch-up fat exhibit normal glucose tolerance, but are hyperinsulinemic as judged by higher plasma insulin concentrations after a glucose load.

### Pancreas perfusions (in situ)

To evaluate insulin-secretory capacity of the endocrine pancreas, refed and control rats were anesthetized with sodium pentothal and prepared for pancreas perfusion as previously described [10]. Briefly, the pancreas was perfused with a Krebs-Hank's buffer (KHB) at a constant rate of 5 ml/min *via *mesenteric and transileac arteries, and the perfusate was collected every minute from a catheter placed in the portal vein. After an initial equilibration period with no sample collected, the effluent was collected in 1-min fractions from the portal vein. The pancreas was perfused at 37°C with the KHB buffer supplemented with the following concentrations of glucose: period I (basal, last 4 min) 2.8 mmol/l glucose, periods II and III (15 min each) 16.7 mmol/l glucose, period IV (recovery, 15 min) 2.8 mmol/l glucose. Aliquots of perfusates were collected on ice and stored at -20°C until insulin assay by radioimmunoassay (RIA) using rat insulin as standard.

### Isolated islet perifusions (ex vivo)

To evaluate the kinetics of insulin secretion in islet-perifusion experiments, pancreatic islets were isolated by collagenase digestion and handpicking from refed and control rats as described previously [11]. Isolated islets were cultured free-floating in RPMI 1640 medium before experiments. Insulin levels were determined by RIA and insulin secretion collected every min was normalized per islet number. Islet perifusions were carried out using 15 to 20 hand-picked islets per chamber of 250 μl volume thermostated at 37°C (Brandel, Gaithersburg, MD, USA). The flux was set at 0.5 ml/min and fractions were collected every min following a 20-min washing period at basal glucose. Rat islets were perifused with Krebs-Ringer bicarbonate HEPES buffer at basal 2.8 mmol/l glucose for 20 min, then stimulated with 8.0 mmol/l glucose (20 min) and 16.7 mmol/l glucose (20 min), returning to 2.8 mmol/l glucose (last 10 min).

### Immunohistochemistry

Pancreata were harvested in cold PBS and treated overnight at 4°C in 4% paraformaldehyde before embedding in paraffin and 5 μm-thick tissue sections were mounted on adhesive-coated slides. Pancreata sections were incubated with a diluted primary antibody for 2 hours at room temperature, and with an appropriate Cy3- (Jackson ImmunoResearch Laboratories, Inc, WestGrove, PA, USA) or ALEXA-conjugated (Molecular Probes, Inc., Eugene, OR, USA) anti IgG serum for 1 hour. The antibodies and their dilution used in the present analysis were as follows: guinea pig anti-insulin (Dako, Carpinteria, CA, USA; dilution 1/400), rabbit anti-glucagon (Dako, Carpinteria, CA, USA; dilution 1/100). Sections were analyzed on a Zeiss Axiophot microscope equipped with an Axiocam color CCD camera (Carl Zeiss, Feldbach, Switzerland).

### Statistics

Data are expressed as mean ± SE, and were analyzed by either unpaired t-test or analysis of variance, using computer software STATISTIK 8 (Analytical Software, St. Paul, Minnesota).

## Results

### Pancreatic perfusions (in situ)

**Figure 2:**
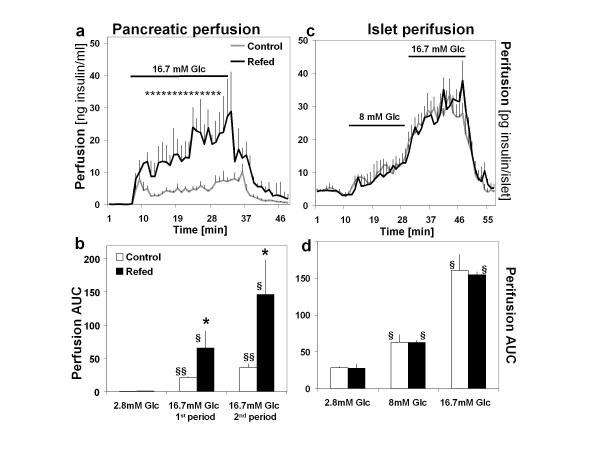
**Kinetics of insulin secretion**. Panel a shows the kinetics of insulin secretion from *in situ *perfusion of pancreas in response to glucose (Glc) in refed and control animals (n = 6) on day 7 of refeeding, first in response to 2.8 mM glucose (baseline), followed by two successive periods lasting 15 min each in response to 16.7 mmol/l glucose before switching back to 2.8 mmol/l glucose. Panel b shows the area under the curve (AUC) for each time-period, calculated after subtraction of basal release. All values are mean ± SE. Symbols for statistical significance of differences are as follows: panel a: * p < 0.05 (at least) between refed and control for corresponding time points; Panel b: * p < 0.05: between-group comparison (refed vs control) for AUC within 1^st ^or 2^nd ^period; **§ **p < 0.05; **§§**p < 0.01: within-group comparison between 16.7 mM Glc or 8 mM relative to basal 2.8 mM Glc. Panel c indicates the kinetics of insulin secretion from *ex vivo *perifusion of islets isolated from pancreases of refed and control groups (n = 5) on day 7 of refeeding, first in response to 2.8 mmol/l glucose (baseline), followed by two successive periods of 17 min with 8 and 16.7 mmol/l glucose, respectively, before switching back to 2.8 mmol/l glucose; panel d shows the AUC for each time-period, calculated after subtraction of basal release. Panel d shows the area under the curve (AUC) for each time-period, calculated after subtraction of basal release All values are mean ± SE. Symbols for statistical significance of differences in panel b are as follows: **§ **p < 0.05: within-group comparison between 16.7 mM Glc or 8 mM Glc relative to basal 2.8 mM Glc

Figure 2 (panel a) shows the profiles of insulin secretion assessed by *in situ *perfusion of intact pancreas from refed and control animals on day 7 of refeeding. Raising glucose (Glc) from 2.8 to 16.7 mmol/l led to a much more pronounced increase in insulin release as a function of time in refed rats than in controls. The area under the curve (AUC), calculated after subtraction of basal release and shown in Figure 2 (panel b), was greater by 3- and 4-fold in the first and second 15 min period respectively, in refed than in control animals (P < 0.01, by Student's *t*-test). During the recovery period (upon shifting back to 2.8 mmol/l glucose), the differences in insulin secretion between the two groups were markedly attenuated and no longer significant.

### Isolated islet perifusions (ex vivo)

The kinetics of insulin secretion in islet-perifusion experiments, shown in Figure 2 (panel c and d) indicated that, once isolated, islets from refed and control animals responded similarly to 8.0 and 16.7 mmol/l glucose.

### Islets and whole pancreas

**Figure 3:**
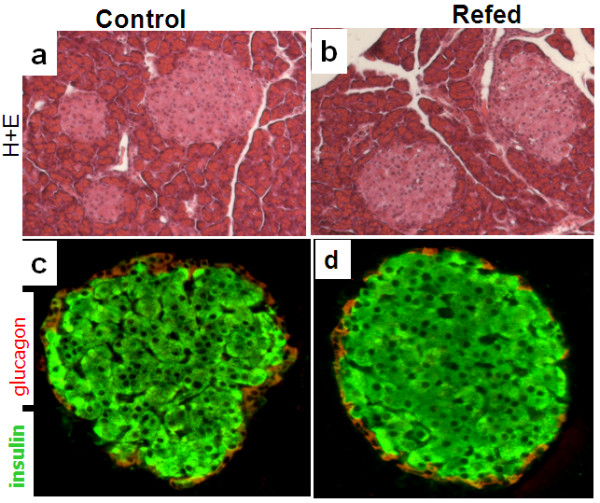
**Pancreatic islet morphology and insulin content**. Panels a and b are pancreata sections stained with hematoxylin and eosin (H+E) showing islets and acinar cells in control and refed rats, respectively. Panel c and d are immunostainings of insulin (in green) and glucagon (in red) showing representative islets from control and refed rats, respectively.

No between-group differences were found in wet weight of fresh pancreases (2.77 ± 0.58 vs 2.49 ± 0.55 g) and in total insulin content (342 ± 55 vs 340 ± 58 μg insulin per g tissue) comparing control and refed animals respectively. Furthermore, immunohistochemistry revealed that islets of refed rats were normal, exhibiting similar beta-cell distribution and size than controls (Figure 3).

### Plasma hormones

No between-group differences were found in plasma concentrations of glucagon-like peptide 1, gastric inhibitory peptide, or leptin (Table 1). By contrast, plasma adiponectin concentrations were higher in the refed animals than in controls (p < 0.01).

**Table 1:** Plasma concentrations of hormones on day 7 of refeeding

	Control	Refed	t-test
Glucagon-like peptide-1 *(ng/ml)*	0.28 ± 0.02	0.24 ± 0.02	NS
Gastric inhibitory peptide *(ng/ml)*	0.49 ± 0.04	0.55 ± 0.07	NS
Leptin *(ng/ml)*	1.91 ± 0.10	2.17 ± 0.18	NS
Adiponectin *(μg/ml)*	7.34 ± 0.41	11.7 ± 1.60	p < 0.01

All values are mean ± SE (n = 6); NS: no significant differences.

The hormones were assayed by commercial ELISA kits from tail blood collected after a 5-6 h fast.

## Discussion

Beta-cell function was investigated in a rat model of semistarvation-refeeding in which a high metabolic efficiency for body fat recovery (i.e., thrifty metabolism driving catch-up fat) is intricately associated with hyperinsulinemia [8]. Data show that the hyperinsulinemic state of catch-up growth is characterized at the beta-cell level by enhanced secretory response to glucose stimulation. No difference was observed between refed and controls in the weight of the pancreas, pancreatic islet morphology or insulin content. Accordingly, pancreatic insulin hypersecretion during catch-up growth cannot be attributed to an increase in beta-cell mass or pancreatic insulin content and hence in functional cells, but rather resides primarily in an *in situ *beta-cell hyperresponsiveness.

Interestingly, such insulin hypersecretion during catch-up growth was observed in the *in situ *pancreatic perfusion preparation, although not in isolated islets. Therefore, hyperresponsiveness cannot be explained by intra-cellular alterations in metabolism-secretion coupling *per se *nor in the insulin exocytosis mechanisms. The observed phenomenon is likely to reside in differential modulation of the secretory response, possibly through negative modulators of insulin secretion being repressed during catch-up growth, resulting in the observed hyperresponsiveness of the pancreatic response to glucose. Such *in situ *islet tuning could be contributed by neuro-hormonal effectors (e.g glucagon-like peptide 1 [12]), paracrine systems (e.g. dopamine [13,14]), or even composition of surrounding fatty acids [15], all these factors being lost once islets are isolated.

The pancreatic insulin hypersecretion during catch-up growth is, however, unlikely be attributed to glucagon-like peptide 1 and gastric inhibitory peptide since these incretins did not differ in refed and control groups in the post-absorptive state (Table 1) nor after a glucose load (data not shown). It is also unlikely to be consequential to excess adiposity and the associated elevation in circulating leptin since our between-group comparison was conducted on day 7 of refeeding, i.e. at a time-point when body fat and plasma leptin in the refed animals had not yet exceeded those of controls (see Figure 1, panel b and Table 1), respectively. Whether our findings of an elevated plasma adiponectin in the refed group versus controls (Table 1) can be implicated in the increased pancreatic hyperresponsiveness to glucose is at present unknown. This is an avenue for further research, particularly in the light of emerging evidence that adiponectin may act directly on pancreatic beta-cells to enhance insulin secretion [16].

**Figure 4:**
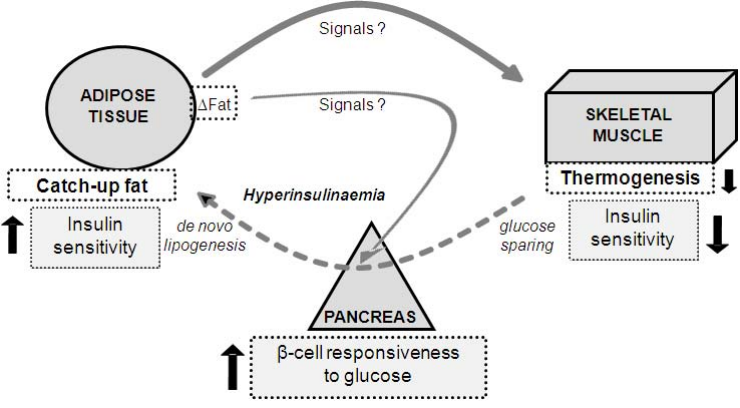
**Model depicting thrifty metabolism underlying catch-up fat and hyperinsulinemia during catch-up growth**. In response to fat depletion and/or delayed fat stores expansion (resulting from energy deficit and growth retardation), energy conservation mechanisms operate via suppressed thermogenesis [8], leading to diminished insulin sensitivity in skeletal muscle [9,17] during increased food availability, so that the spared energy leads to accelerated replenishment of the fat stores (catch-up fat) in adipose tissue. The glucose spared from utilization in skeletal muscle is thus redirected to an adipose tissue that shows increased insulin hyperresponsiveness [9] and enhanced lipogenic machinery [18], under orchestration by hyperinsulinemia which is sustained by pancreatic β-cell hyperresponsiveness (as reported here). In this model, the 'adipostat' signals (?) that dictate suppress thermogenesis and insulin resistance in skeletal muscle are postulated to also dictate the pancreatic beta-cell hyperresponsiveness that will sustain glucose redistribution between skeletal muscle and adipose tissue, thereby contributing to the thrifty 'catch-up fat' phenotype associated with hyperinsulinemia.

Whatever the mechanisms that lead to such beta-cell hyperresponsiveness to glucose during catch-up growth, its demonstration in a rat model in which catch-up fat is driven solely by suppressed thermogenesis (and not hyperphagia) suggests a role for pancreatic islets in the thrifty mechanisms that drive catch-up fat through glucose redistribution between skeletal muscle and adipose tissue [8,9,17,18]. This is depicted in a conceptual model presented in the Figure 4.

An enhanced beta-cell function, as evidenced by an increased insulin release in response to glucose stimulation, has been observed early in the pathogenesis of type 2 diabetes in animal models [19-21]. It has also been shown to be an early characteristic of ethnic groups and people with normal glucose tolerance at higher risks for diabetes [22-27], and is embodied in the concept that β-cell hyperfunction is an early stage in the progression to β-cell failure [28]. The pancreatic β-cell hyperresponsiveness to glucose during catch-up fat may therefore be a key component in the link between catch-up growth and later risks for type 2 diabetes.

## Competing interests

The authors declare that they have no competing interests.

## Authors' contributions

MC researched data, contributed to discussion and reviewed/edited manuscript. PMdA researched data, contributed to discussion and reviewed/edited manuscript. AG researched data. JPM contributed to discussion and reviewed/edited manuscript. PM designed the study, contributed to discussion and wrote manuscript; AGD designed the study, contributed to discussion and wrote manuscript. All authors read and approved the final manuscript.
